# 1-Ethyl-1*H*-2,1-benzothia­zin-4(3*H*)-one 2,2-dioxide

**DOI:** 10.1107/S1600536808003504

**Published:** 2008-02-06

**Authors:** Muhammad Shafiq, Islam Ullah Khan, M. Nawaz Tahir, Waseeq Ahmad Siddiqui

**Affiliations:** a Government College University, Department of Chemistry, Lahore, Pakistan; b Department of Physics, University of Sargodha, Sargodha, Pakistan; c Department of Chemistry, University of Sargodha, Sargodha, Pakistan

## Abstract

In the title compound, C_10_H_11_NO_3_S, there is distorted tetra­hedral geometry around the S atom. The heterocyclic thia­zine ring adopts a half-chair conformation. The ethyl and sulfonyl groups form dihedral angles of 82.53 (13) and 88.91 (9)°, respectively, with the plane formed by the benzothia­zine ring, excluding the S atom; the S atom and the ethyl group lie on opposite sides of the ring. The mol­ecules are linked into dimers by inter­molecular C—H⋯O hydrogen bonds involving benzene C—H and carbonyl O atoms, thus forming eight-membered rings. The dimers are linked into chains *via* inter­actions of a similar type. There is an intra­molecular C—H⋯O hydrogen bond.

## Related literature

For related literature, see: Hanson *et al.* (1999 ▶); Misu & Togo (2003 ▶); Shafiq *et al.* (2008 ▶); Siddique *et al.* (2006 ▶); Siddiqui *et al.* (2007 ▶); Tahir *et al.* (2008 ▶); Cremer & Pople (1975 ▶).
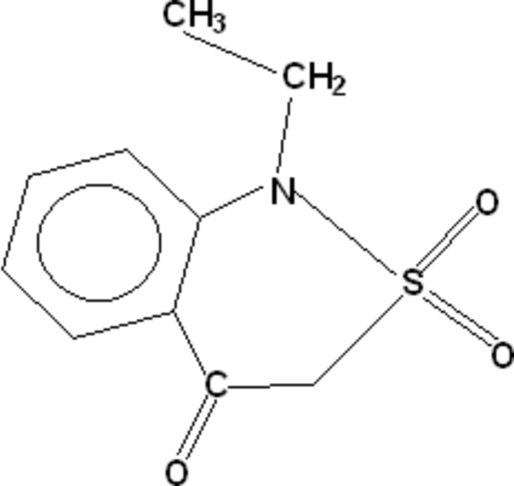


## Experimental

### 

#### Crystal data

C_10_H_11_NO_3_S
*M* *_r_* = 225.26
Triclinic, 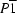

*a* = 7.0272 (3) Å
*b* = 8.0448 (4) Å
*c* = 9.5880 (4) Å
α = 99.124 (3)°
β = 95.075 (3)°
γ = 104.092 (3)°
*V* = 514.48 (4) Å^3^
*Z* = 2
Mo *K*α radiation
μ = 0.30 mm^−1^
*T* = 296 (2) K
0.15 × 0.12 × 0.10 mm

#### Data collection

Bruker Kappa APEXII CCD diffractometer
Absorption correction: multi-scan (*SADABS*; Bruker, 2005 ▶) *T*_min_ = 0.965, *T*_max_ = 0.988
11339 measured reflections
2608 independent reflections
1760 reflections with *I* > 2σ(*I*)
*R*_int_ = 0.036

#### Refinement

*R*[*F* ^2^ > 2σ(*F* ^2^)] = 0.043
*wR*(*F* ^2^) = 0.107
*S* = 1.02
2608 reflections
136 parameters
H-atom parameters constrained
Δρ_max_ = 0.29 e Å^−3^
Δρ_min_ = −0.26 e Å^−3^

### 

Data collection: *APEX2* (Bruker, 2007 ▶); cell refinement: *APEX2*; data reduction: *SAINT* (Bruker, 2007 ▶); program(s) used to solve structure: *SHELXS97* (Sheldrick, 2008 ▶); program(s) used to refine structure: *SHELXL97* (Sheldrick, 2008 ▶); molecular graphics: *ORTEP-3 for Windows* (Farrugia, 1997 ▶) and *PLATON* (Spek, 2003 ▶); software used to prepare material for publication: *WinGX* (Farrugia, 1999 ▶) and *PLATON*.

## Supplementary Material

Crystal structure: contains datablocks global, I. DOI: 10.1107/S1600536808003504/pv2067sup1.cif

Structure factors: contains datablocks I. DOI: 10.1107/S1600536808003504/pv2067Isup2.hkl

Additional supplementary materials:  crystallographic information; 3D view; checkCIF report

## Figures and Tables

**Table 1:** Hydrogen-bond geometry (Å, °)

*D*—H⋯*A*	*D*—H	H⋯*A*	*D*⋯*A*	*D*—H⋯*A*
C2—H2⋯O3^i^	0.93	2.56	3.478 (3)	169
C8—H8*A*⋯O1^ii^	0.97	2.50	3.388 (3)	152
C9—H9*B*⋯O2	0.97	2.36	2.855 (3)	111

Symmetry codes: (i) 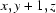; (ii) 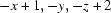.

**Fig. 1.**
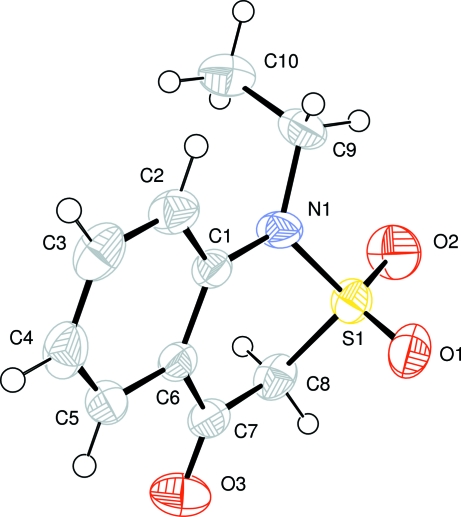
ORTEP-3 (Farrugia, 1997) drawing of (I) with the atom numbering scheme. The thermal ellipsoids are drawn at the 50% probability level.

**Fig. 2.**
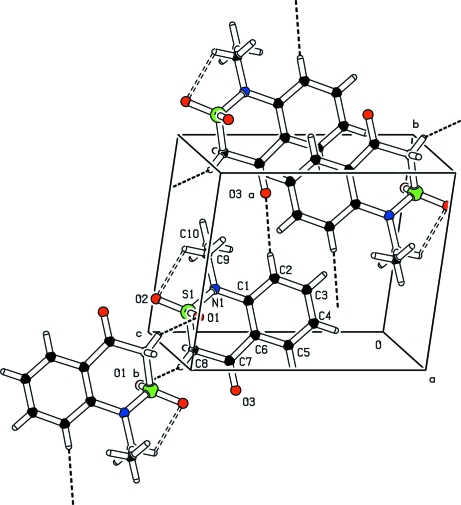
The unit cell packing of (I) (Spek, 2003) showing the intermolecular hydrogen bonds resulting in dimers.

## supplementary crystallographic information

### Comment

Sulfonamides in general, and cyclic sulfonamides (sultams) in particular are important therapeutic compounds (Hanson *et al.*, 1999). Among sultams, 1,2-benzothiazine and 2,1-benzothiazine dioxides (benzosultams) have proven to be biologically active (Misu & Togo, 2003). Due to the importance of 2,1-benzothiazine derivatives in medicinal chemistry, their synthesis has gained enormous attention. After accomplishing the synthesis of a number of 1,2-benzothiazine 1,1-dioxide derivatives (Siddique *et al.*, 2006 and Siddiqui *et al.*, 2007), we have recently started the synthesis of various 2,1-benzothiazine 2,2-dioxide derivatives.

The title compound, (I), was synthesized in continuation to our research on derivatives of 2,1-benzothiazine. It is a cyclized product of methyl 2-(*N*-ethylmethanesulfonamido)benzoate (Shafiq *et al.*, 2008). The hetrocyclic ring adopts a half chair confirmation which may be described by the puckering parameters (Cremer & Pople, 19975): Q = 0.554 (2) Å, θ = 53.6 (2)° and φ = 356.2 (3)°. The structure of (I) can be best compared with its 1-methyl analogue (Tahir *et al.*, 2008). In (I), the bond distance N1—C9 [1.477 (2) Å] is significantly longer than the corresponding distance [1.452 (2) Å] in the 1-methyl analogue. The range of bond angles around S in the two structures are essentially identical. All the atoms in the benzothiazine ring in (I) are nearly planer except that of S1 which is displaced by 0.783 (2) Å from the plane defined by C1—C8/N1, while C9-atom of *N*-ethyl group is at a distance of -0.226 (3) Å. The *N*-ethyl and sulfonyl groups form dihedral angles of 82.53 (13)° and 88.91 (9)°, respectively, with the plane formed by C1—C8/N1 atoms. The dihedral angle between these two groups is 46.66 (5)°. In the asymmetric unit there is an intramolecular H-bond between C9 and O2 atoms. The molecules are dimerized by forming eight member rings through H-bonding between methylene group of thiazine ring and sulfonyl O-atom (C8—H8···O1). The structure is further stabilized by interactions involving phenyl C—H and carbonyl O-atoms (C2—H2···O3) linking dimers into chains. Fig. 2 shows hydrogen bonding interactions; details of H-bonding geometry are given in Table 1.

### Experimental

A suspension of hexane-washed sodium hydride (4.6 g, 96.0 mmol., 50% in mineral oil) was prepared in dry dimethylformamide (30 ml). To this suspension, a solution of methyl 2-(*N*-ethylmethanesulfonamido)benzoate (19.02 g, 74.0 mmol) in dry dimethylformamide (70 ml) was added. The reaction mixture was stirred at room temperature (1.5 h) and was poured in a thin stream into hydrochloric acid (3 N, 200 ml). The pH of the mixture was then adjusted to neutral using NaHCO_3_. After this it was filtered and the filtrate was evaporated under reduced pressure (11 torr) to obtain the title compound (yield; 15 g, 90%); m.p. 354–355 K. Colorless crystals of (I) suitable for X-ray diffraction were grown from MeOH by slow evaporation at room temperature.

### Refinement

H atoms were positioned geometrically, with C—H = 0.93, 0.97, and 0.96 Å for aromatic, methylene and methyl H, and constrained to ride on their parent atoms, with *U*_iso_(H) = *xU*_eq_(C), where *x* = 1.5 for methyl H, and *x* = 1.2 for all other H atoms.

### Crystal data

**Table:**

C_10_H_11_NO_3_S	*Z* = 2
*M**_r_* = 225.26	*F*_000_ = 236
Triclinic, *P*1	*D*_x_ = 1.454 Mg m^−^^3^
Hall symbol: -P 1	Mo *K*α radiation λ = 0.71073 Å
*a* = 7.0272 (3) Å	Cell parameters from 1760 reflections
*b* = 8.0448 (4) Å	θ = 2.2–28.7º
*c* = 9.5880 (4) Å	µ = 0.30 mm^−^^1^
α = 99.124 (3)º	*T* = 296 (2) K
β = 95.075 (3)º	Prismatic, colourless
γ = 104.092 (3)º	0.15 × 0.12 × 0.10 mm
*V* = 514.48 (4) Å^3^	

### Data collection

**Table:**

Bruker Kappa APEXII CCD diffractometer	2608 independent reflections
Radiation source: fine-focus sealed tube	1760 reflections with *I* > 2σ(*I*)
Monochromator: graphite	*R*_int_ = 0.036
Detector resolution: 7.40 pixels mm^-1^	θ_max_ = 28.7º
*T* = 296(2) K	θ_min_ = 2.2º
ω scans	*h* = −9→9
Absorption correction: multi-scan(SADABS; Bruker, 2005)	*k* = −10→10
*T*_min_ = 0.965, *T*_max_ = 0.988	*l* = −12→12
11339 measured reflections	

### Refinement

**Table:**

Refinement on *F*^2^	Secondary atom site location: difference Fourier map
Least-squares matrix: full	Hydrogen site location: inferred from neighbouring sites
*R*[*F*^2^ > 2σ(*F*^2^)] = 0.043	H-atom parameters constrained
*wR*(*F*^2^) = 0.107	*w* = 1/[σ^2^(*F*_o_^2^) + (0.0488*P*)^2^ + 0.0854*P*] where *P* = (*F*_o_^2^ + 2*F*_c_^2^)/3
*S* = 1.02	(Δ/σ)_max_ < 0.001
2608 reflections	Δρ_max_ = 0.29 e Å^−^^3^
136 parameters	Δρ_min_ = −0.26 e Å^−^^3^
Primary atom site location: structure-invariant direct methods	Extinction correction: none

### Special details

**Table:**

Geometry. All e.s.d.'s (except the e.s.d. in the dihedral angle between two l.s. planes) are estimated using the full covariance matrix. The cell e.s.d.'s are taken into account individually in the estimation of e.s.d.'s in distances, angles and torsion angles; correlations between e.s.d.'s in cell parameters are only used when they are defined by crystal symmetry. An approximate (isotropic) treatment of cell e.s.d.'s is used for estimating e.s.d.'s involving l.s. planes.
Refinement. Refinement of *F*^2^ against ALL reflections. The weighted *R*-factor *wR* and goodness of fit *S* are based on *F*^2^, conventional *R*-factors *R* are based on *F*, with *F* set to zero for negative *F*^2^. The threshold expression of *F*^2^ > σ(*F*^2^) is used only for calculating *R*-factors(gt) *etc*. and is not relevant to the choice of reflections for refinement. *R*-factors based on *F*^2^ are statistically about twice as large as those based on *F*, and *R*- factors based on ALL data will be even larger.

### Fractional atomic coordinates and isotropic or equivalent isotropic displacement parameters (Å^2^)

**Table:**

	*x*	*y*	*z*	*U*_iso_*/*U*_eq_	
S1	0.32887 (8)	0.16467 (6)	0.90938 (5)	0.04169 (17)	
O1	0.5349 (2)	0.18066 (19)	0.90570 (14)	0.0531 (4)	
O2	0.2664 (3)	0.2196 (2)	1.04236 (14)	0.0670 (5)	
O3	0.2248 (2)	−0.25167 (19)	0.63616 (17)	0.0632 (4)	
N1	0.2390 (2)	0.2638 (2)	0.79263 (15)	0.0415 (4)	
C1	0.2558 (3)	0.2075 (2)	0.64688 (17)	0.0328 (4)	
C2	0.2726 (3)	0.3249 (3)	0.5533 (2)	0.0429 (5)	
H2	0.2746	0.4406	0.5861	0.051*	
C3	0.2864 (3)	0.2688 (3)	0.4118 (2)	0.0489 (5)	
H3	0.2959	0.3475	0.3498	0.059*	
C4	0.2862 (3)	0.1000 (3)	0.3604 (2)	0.0485 (5)	
H4	0.2982	0.0651	0.2651	0.058*	
C5	0.2684 (3)	−0.0161 (3)	0.45077 (19)	0.0420 (5)	
H5	0.2679	−0.1310	0.4160	0.050*	
C6	0.2509 (2)	0.0337 (2)	0.59449 (17)	0.0329 (4)	
C7	0.2276 (3)	−0.1022 (2)	0.6829 (2)	0.0389 (4)	
C8	0.2024 (3)	−0.0522 (3)	0.8374 (2)	0.0445 (5)	
H8A	0.2512	−0.1285	0.8919	0.053*	
H8B	0.0627	−0.0687	0.8454	0.053*	
C9	0.2066 (3)	0.4367 (3)	0.8431 (2)	0.0461 (5)	
H9A	0.2951	0.5239	0.8032	0.055*	
H9B	0.2378	0.4664	0.9461	0.055*	
C10	−0.0036 (3)	0.4394 (3)	0.8011 (3)	0.0575 (6)	
H10A	−0.0197	0.5532	0.8354	0.086*	
H10B	−0.0914	0.3545	0.8420	0.086*	
H10C	−0.0341	0.4120	0.6992	0.086*	

### Atomic displacement parameters (Å^2^)

**Table:**

	*U*^11^	*U*^22^	*U*^33^	*U*^12^	*U*^13^	*U*^23^
S1	0.0517 (3)	0.0444 (3)	0.0303 (2)	0.0137 (2)	0.00127 (19)	0.01149 (19)
O1	0.0444 (9)	0.0558 (9)	0.0541 (8)	0.0071 (7)	−0.0081 (6)	0.0135 (7)
O2	0.0971 (13)	0.0800 (12)	0.0318 (7)	0.0339 (10)	0.0142 (8)	0.0136 (7)
O3	0.0782 (12)	0.0343 (9)	0.0810 (11)	0.0192 (8)	0.0145 (9)	0.0127 (7)
N1	0.0633 (11)	0.0387 (9)	0.0299 (7)	0.0260 (8)	0.0075 (7)	0.0082 (6)
C1	0.0347 (10)	0.0366 (10)	0.0302 (8)	0.0141 (8)	0.0044 (7)	0.0083 (7)
C2	0.0504 (12)	0.0408 (11)	0.0434 (10)	0.0167 (9)	0.0083 (9)	0.0163 (8)
C3	0.0492 (13)	0.0673 (15)	0.0394 (10)	0.0197 (11)	0.0106 (9)	0.0273 (10)
C4	0.0436 (12)	0.0748 (16)	0.0305 (9)	0.0224 (10)	0.0055 (8)	0.0083 (9)
C5	0.0374 (11)	0.0459 (12)	0.0407 (10)	0.0152 (9)	0.0000 (8)	−0.0022 (8)
C6	0.0297 (9)	0.0366 (11)	0.0337 (9)	0.0118 (8)	0.0026 (7)	0.0066 (7)
C7	0.0332 (10)	0.0337 (11)	0.0498 (11)	0.0092 (8)	0.0013 (8)	0.0095 (8)
C8	0.0446 (12)	0.0431 (12)	0.0485 (11)	0.0080 (9)	0.0024 (9)	0.0237 (9)
C9	0.0618 (14)	0.0334 (11)	0.0423 (10)	0.0152 (10)	0.0087 (9)	−0.0004 (8)
C10	0.0615 (15)	0.0474 (14)	0.0693 (14)	0.0239 (11)	0.0180 (11)	0.0075 (11)

### Geometric parameters (Å, °)

**Table:**

S1—O2	1.4244 (14)	C4—H4	0.9300
S1—O1	1.4260 (14)	C5—C6	1.398 (2)
S1—N1	1.6405 (15)	C5—H5	0.9300
S1—C8	1.750 (2)	C6—C7	1.473 (2)
O3—C7	1.210 (2)	C7—C8	1.510 (3)
N1—C1	1.424 (2)	C8—H8A	0.9700
N1—C9	1.477 (2)	C8—H8B	0.9700
C1—C2	1.395 (2)	C9—C10	1.503 (3)
C1—C6	1.400 (2)	C9—H9A	0.9700
C2—C3	1.380 (3)	C9—H9B	0.9700
C2—H2	0.9300	C10—H10A	0.9600
C3—C4	1.368 (3)	C10—H10B	0.9600
C3—H3	0.9300	C10—H10C	0.9600
C4—C5	1.363 (3)		
			
O2—S1—O1	118.03 (10)	C5—C6—C1	119.07 (16)
O2—S1—N1	107.40 (9)	C5—C6—C7	117.38 (17)
O1—S1—N1	111.71 (9)	C1—C6—C7	123.54 (16)
O2—S1—C8	110.60 (10)	O3—C7—C6	122.76 (18)
O1—S1—C8	107.58 (9)	O3—C7—C8	119.04 (17)
N1—S1—C8	100.05 (8)	C6—C7—C8	118.20 (16)
C1—N1—C9	121.18 (14)	C7—C8—S1	112.13 (13)
C1—N1—S1	117.15 (12)	C7—C8—H8A	109.2
C9—N1—S1	118.59 (12)	S1—C8—H8A	109.2
C2—C1—C6	119.03 (16)	C7—C8—H8B	109.2
C2—C1—N1	120.08 (16)	S1—C8—H8B	109.2
C6—C1—N1	120.87 (15)	H8A—C8—H8B	107.9
C3—C2—C1	119.70 (18)	N1—C9—C10	111.54 (17)
C3—C2—H2	120.2	N1—C9—H9A	109.3
C1—C2—H2	120.2	C10—C9—H9A	109.3
C4—C3—C2	121.64 (18)	N1—C9—H9B	109.3
C4—C3—H3	119.2	C10—C9—H9B	109.3
C2—C3—H3	119.2	H9A—C9—H9B	108.0
C5—C4—C3	119.13 (18)	C9—C10—H10A	109.5
C5—C4—H4	120.4	C9—C10—H10B	109.5
C3—C4—H4	120.4	H10A—C10—H10B	109.5
C4—C5—C6	121.41 (18)	C9—C10—H10C	109.5
C4—C5—H5	119.3	H10A—C10—H10C	109.5
C6—C5—H5	119.3	H10B—C10—H10C	109.5
			
O2—S1—N1—C1	−170.18 (14)	C4—C5—C6—C7	178.61 (17)
O1—S1—N1—C1	58.92 (16)	C2—C1—C6—C5	1.7 (3)
C8—S1—N1—C1	−54.70 (16)	N1—C1—C6—C5	−179.84 (16)
O2—S1—N1—C9	29.41 (18)	C2—C1—C6—C7	−178.16 (17)
O1—S1—N1—C9	−101.49 (15)	N1—C1—C6—C7	0.3 (3)
C8—S1—N1—C9	144.89 (15)	C5—C6—C7—O3	0.5 (3)
C9—N1—C1—C2	9.9 (3)	C1—C6—C7—O3	−179.56 (17)
S1—N1—C1—C2	−149.95 (15)	C5—C6—C7—C8	−178.23 (15)
C9—N1—C1—C6	−168.48 (17)	C1—C6—C7—C8	1.7 (3)
S1—N1—C1—C6	31.6 (2)	O3—C7—C8—S1	149.47 (16)
C6—C1—C2—C3	−0.7 (3)	C6—C7—C8—S1	−31.7 (2)
N1—C1—C2—C3	−179.18 (17)	O2—S1—C8—C7	166.56 (13)
C1—C2—C3—C4	−0.8 (3)	O1—S1—C8—C7	−63.23 (15)
C2—C3—C4—C5	1.2 (3)	N1—S1—C8—C7	53.53 (15)
C3—C4—C5—C6	−0.2 (3)	C1—N1—C9—C10	75.8 (2)
C4—C5—C6—C1	−1.3 (3)	S1—N1—C9—C10	−124.64 (16)

### Hydrogen-bond geometry (Å, °)

**Table:**

*D*—H···*A*	*D*—H	H···*A*	*D*···*A*	*D*—H···*A*
C2—H2···O3^i^	0.93	2.56	3.478 (3)	169
C8—H8A···O1^ii^	0.97	2.50	3.388 (3)	152
C9—H9B···O2	0.97	2.36	2.855 (3)	111

Symmetry codes: (i) *x*, *y*+1, *z*; (ii) −*x*+1, −*y*, −*z*+2.

## References

[bb1] Bruker (2005). *SADABS* Bruker AXS Inc., Madison, Wisconsin, USA.

[bb2] Bruker (2007). *APEX2* (Version 1.27) and *SAINT* (Version 7.12a). Bruker AXS Inc., Madison, Wisconsin, USA.

[bb3] Cremer, D. & Pople, J. A. (1975). *J. Am. Chem. Soc.* **97**, 1354–1358.

[bb4] Farrugia, L. J. (1997). *J. Appl. Cryst.* **30**, 565.

[bb5] Farrugia, L. J. (1999). *J. Appl. Cryst.* **32**, 837–838.

[bb6] Hanson, P. R., Probst, D. A., Robinson, R. E. & Yau, M. (1999). *Tetrahedron Lett.* **40**, 4761–4764.

[bb7] Misu, Y. & Togo, H. (2003). *Org. Biomol. Chem.* **1**, 1342–1346. 10.1039/b301330h 12929664

[bb8] Shafiq, M., Tahir, M. N., Khan, I. U., Siddiqui, W. A. & Arshad, M. N. (2008). *Acta Cryst.* E**64**, o389. 10.1107/S160053680800007X PMC2960301 21201419

[bb9] Sheldrick, G. M. (2008). *Acta Cryst.* A**64**, 112–122. 10.1107/S0108767307043930 18156677

[bb10] Siddique, W. A., Ahmad, S., Khan, I. U. & Malik, A. (2006). *J. Chem. Soc. Pak.* **28**, 583–589.

[bb11] Siddiqui, W. A., Ahmad, S., Khan, I. U., Siddiqui, H. L. & Weaver, G. W. (2007). *Synth. Commun.* **37**, 767–773.

[bb12] Spek, A. L. (2003). *J. Appl. Cryst.* **36**, 7–13.

[bb13] Tahir, M. N., Shafiq, M., Khan, I. U., Siddiqui, W. A. & Arshad, M. N. (2008). *Acta Cryst.* E**64**, o557. 10.1107/S1600536808003498 PMC2960778 21201900

